# Impact of Parenteral Prostanoids in Pulmonary Arterial Hypertension: The Relevance of Timing

**DOI:** 10.3390/jcm12216840

**Published:** 2023-10-29

**Authors:** Silvia Papa, Gianmarco Scoccia, Giorgia Serino, Francesca Ileana Adamo, Jean Pierre Jabbour, Annalisa Caputo, Michela Boromei, Domenico Filomena, Domenico Laviola, Enrico Maggio, Giovanna Manzi, Alexandra Mihai, Tommaso Recchioni, Alexandra Sabusco, Livia Valeri, Sara Vinciullo, Carmine Dario Vizza, Roberto Badagliacca

**Affiliations:** 1 Department of Clinical, Internal Medicine, Anesthesiology and Cardiovascular Sciences, Sapienza University of Rome, 00161 Rome, Italy; gianmarcoscoccia@gmail.com (G.S.); giorgia.serino@uniroma1.it (G.S.); francescaileana.adamo@uniroma1.it (F.I.A.); jpj1995@gmail.com (J.P.J.); annalisa.caputo@uniroma1.it (A.C.); michela.boromei@uniroma1.it (M.B.); domenico.filomena@uniroma1.it (D.F.); domenico.laviola@uniroma1.it (D.L.); enrico.maggio@uniroma1.it (E.M.); giovannamanzi91@gmail.com (G.M.); mihai.1730169@studenti.uniroma1.it (A.M.); tommaso.recchioni@uniroma1.it (T.R.); liviavaleri20@gmail.com (L.V.); sara.vinciullo@uniroma1.it (S.V.); dario.vizza@uniroma1.it (C.D.V.); roberto.badagliacca@uniroma1.it (R.B.); 2 Department of Translational Medicine, University of Eastern Piedmont, 28100 Novara, Italy; alesabusco@yahoo.it

**Keywords:** pulmonary arterial hypertension, pulmonary vascular resistance, reverse remodeling, prostanoids

## Abstract

Parenteral prostanoids are being recommended in pulmonary arterial hypertension (PAH) treatment, but the prognostic relevance of delayed treatment initiation is still debated. This study assessed the impact of the timing of prostacyclin treatment initiation on reducing PVR and achieving a low-risk profile in PAH patients. The study enrolled 151 patients who started on parenteral prostanoids with different treatment strategies. All patients underwent right heart catheterization, clinical evaluation, and risk assessments at baseline and after 1-year follow-up. Patients with an upfront strategy including parenteral prostanoid plus one oral drug had −5.3 ± 6.2 WU (−50 ± 19%) reduction in PVR, patients with an upfront strategy including parenteral prostanoid plus double oral drug had −12.8 ± 5.9 WU (−68 ± 17%) reduction in PVR, while patients with an add-on strategy including parenteral prostanoid after oral drugs had −3.9 ± 3.5 WU (−23 ± 19%) reduction in PVR. An upfront strategy including parenteral prostanoids was independently associated with an increased likelihood of achieving the greater reduction of PVR compared with an add-on strategy. Additionally, the greater the severity of PH at the time of diagnosis, in terms of PVR and RV reverse remodeling, the higher the probability of treatment failure. An upfront strategy including a parenteral prostanoid is associated with the highest likelihood of achieving a low-risk profile and a greater reduction of PVR compared with parenteral prostanoid as an add-on to oral treatment.

## 1. Introduction

The PH ESC/ERS Guidelines recommend upfront double oral therapy for low- and intermediate-risk patients [1]. This approach has been recently confirmed in the PH World Symposium 2018 [2]. The expert panel proposal was to evaluate risk assessment and therefore treatment strategy mainly based on WHO class, 6MWT, cardiac index, and BNP (average method or number of low-risk variables present) [3,4,5]. However, recent data from the Italian network (iPHNET) [6] show the insufficient power of the oral upfront treatment to achieve a low-risk condition, for a significant proportion of intermediate-risk patients. The results demonstrate that less than 40% of intermediate-risk PAH naïve patients treated with upfront double oral therapy are able to achieve a low-risk status (whatever the risk score considered—European or REVEAL 2.0) mainly because of the poor hemodynamic response (in terms of PVR reduction) for the majority of patients. Interestingly the results show that a low-risk status is tightly related to a large reduction in PVR, that is achieved only in approx. 30% of treated patients.

The determinants of a poor hemodynamic response have been identified in the study with an age > 60, the male sex, an mPAP > 48 mmHg (associated to CI < 2.5), and an RV/LV area ratio > 1 (associated to TAPSE < 18 mm), resulting independent determinants [6].

These results highlight our clinical need to identify, particularly among the intermediate risk group, those patients who should be treated with upfront therapy including parenteral prostanoids [7].

Our group has also already shown the prognostic importance of achieving a reverse remodeling of the RV among other clinical and hemodynamic parameters (WHO class, 6MWT, RAP, CI) and the importance of reducing PVR for RV reverse remodeling. The Italian network (iPHNET) has also shown [8] that the best way to reach a low-risk status seems to probably achieve a huge PVR reduction that will allow significant right ventricular improvement. This goal is more easily achieved with more aggressive strategies including parenteral prostanoids [9].

The aim of the present study was to investigate the impact of the timing of prostacyclin treatment initiation on reducing PVR and achieving a low-risk profile in PAH patients treated with different treatment strategies.

## 2. Materials and Methods

### 2.1. Study Population

One hundred-fifty-one consecutive PAH patients referred to our center from January 1998 to December 2019 were enrolled in the study if treated with parenteral prostanoids. Patients were started on parenteral prostanoid with different treatment strategies: upfront parenteral prostanoid plus one oral drug (ERA or PD5i), upfront parenteral prostanoid plus two oral drugs (ERA and PD5i), and add-on parenteral prostanoid to oral drugs (monotherapy or double oral at diagnosis). Epoprostenol i.v. and Treprostinil s.c. (from 2006) were the parenteral prostanoids used in our center. 

The diagnostic work-up of PAH was conducted in accordance with the hemodynamic definition of the European guidelines in force at the time of the population’s enrollment: mean pulmonary artery pressure (mPAP) ≥25 mmHg, pulmonary artery wedge pressure <15 mm Hg, and PVR ≥3 Wood units (WU), and the use of an algorithm including respiratory function tests, perfusion lung scan, computed tomographic scan, and echocardiography. All patients were non-responders to acute vasodilator testing with nitric oxide at the time of diagnosis.

Baseline evaluation included demographics, medical history, physical examination, a non-encouraged 6-min walk test (6MWT), right heart catheterization (RHC), and echocardiographic evaluation (Vivid S6; GE Medical Systems). The complete assessment was repeated at a mean of 12 months from baseline (diagnosis for naïve patients starting upfront parenteral prostanoids; time starting add-on parenteral prostanoids for prevalent patients). This second assessment was performed before any additional therapy on top of the initial upfront or add-on strategy to avoid confounding factors, as part of our clinical practice.

From 1998 to date, it has been our strategy to continuously increase the dose of prostanoids to the maximum tolerated dose. High cardiac output was evaluated by echocardiography determined measurements to avoid adverse consequences [8].

Prostanoid was started at a dose of 2 ng/kg/min and increased to achieve approximately 10 ng/kg/min at hospital discharge (1–2 weeks). It was additionally increased on an outpatient basis by 1–2 ng/kg/min weekly for 6 months and then by 2 ng/kg/min every 1–2 months. Clinical improvement in the WHO class and 6MWT were not considered reasons not to further increase dosage.

From 2006 onwards, epoprostenoli.v. and treprostinils.c. were chosen based on patient compliance and the need for a prompter clinical response for i.v. administration.

The study complies with the Declaration of Helsinki and was approved by our local Institutional Review Boards for human studies (Protocol Ref. 5361).

### 2.2. Risk Assessment

Risk assessment was based on a simplified version of the ERS/ESC guidelines’ score, with incorporation of WHO functional class, 6-min walk distance (6MWD), right atrial pressure (RAP), and cardiac index (CI) [5].

### 2.3. Right Heart Catheterization

Hemodynamic evaluation was carried out with the standard technique. Pressures were measured from the mid-chest position with a fluid-filled catheter and pressure transducer, recording the average values over three respiratory cycles, according to a common protocol highlighted by guidelines. Cardiac output (CO) was measured by the thermodilution technique (American Edwards Laboratories, Santa Ana, CA, USA) and pulmonary vascular resistance (PVR) was calculated with the formula PVR = (mPAP-PWP)/CO.

### 2.4. Statistical Analysis

Continuous data are expressed as mean ± standard deviation, while not normally distributed variables are reported as medians and interquartile ranges (IQR). Categorical data are expressed as counts and proportions. Two-group comparisons were carried out with unpaired or paired, two-tailed t tests for means if the data were normally distributed or with Wilcoxon’s rank-sum tests if the data were not normally distributed. Chi square or Fisher’s exact tests were used to analyze the categorical data.

Linear regression analysis was performed to assess the relations between continuous variables and expressed as a Pearson correlation coefficient.

Whenever the data were not belonging to normal distributions or were categorical, the following tests were used to assess the relations between variables: non-parametric Spearman correlation method, which yields the rank correlation coefficient (rho); analysis of categorical data (contingency tables) by chi-square test, with odds ratio calculation; McNemar test for proportions using paired samples; three-way contingency tables with the Cochran–Mantel–Haenszel test for comparing proportions.

Logistic regression analysis was used to identify the following:

- The determinants of the higher tertile of PVR reduction;
- The determinants of the achievement of a low-risk profile at second observation (12 ± 6 months from diagnosis).

All statistical analyses were performed using SPSS software (version 26.0, IBM Corp., Armonk, NY, USA). All statistical tests were two-sided, and a *p* value < 0.05 was considered statistically significant.

## 3. Results

### 3.1. Study Population

One hundred-fifty-one consecutive PAH patients referred to our center were considered in the present study. Table 1 summarizes the baseline characteristics of the study population at the time of diagnosis.

The majority of patients had idiopathic pulmonary arterial hypertension (106, 70.2%), 26 (17.2%) patients had CTD PAH, 3 (2.0%) patients had HIV PAH, 6 (4.0%) patients had corrected-CHD PAH, and 10 (6.6%) patients had portopulmonary hypertension.

The patients were predominantly females (71.5%) with a mean age of 55 ± 14 years. The majority of them were in WHO functional class III, with severe pulmonary hypertension and impaired functional capacity.

According to the ERS/ESC guidelines’ risk assessment, 131 (86.8%) of the patients were at intermediate risk, and 20 (13.2%) were at high risk at the time of diagnosis.

Twenty-five (16.6%) patients were started on parenteral prostanoid monotherapy, 26 (17.2%) patients on upfront treatment with one oral drug (ERA or PD5i) plus parenteral prostanoid, 28 (18.5%) patients on upfront triple combination including parenteral prostanoid, and 72 (47.7%) patients started a parenteral prostanoid during follow-up as an add-on treatment after oral therapy (ERA or PD5i monotherapy or ERA and PD5i).

Side effects of PAH treatment were mild and in accordance with those already known from randomized controlled trials: diarrhea, flushing, nausea, hypotension, peripheral edema and site pain or infusion site reaction and jaw pain (in patients with prostanoids). Notably, none of the patients needed to withdraw the treatment regimen.

### 3.2. Hemodynamic Changes at Follow-Up and Determinants of PVR Reduction

All the patients survived after a mean of 383 ± 560 days follow-up. As shown in Table 2, the patients had significant improvements in WHO functional class, 6MWD, PVR, mPAP, RAP, CI, RVEDA, RA area, RVFAC, TAPSE, and tricuspid regurgitation.

**Figure 1:**
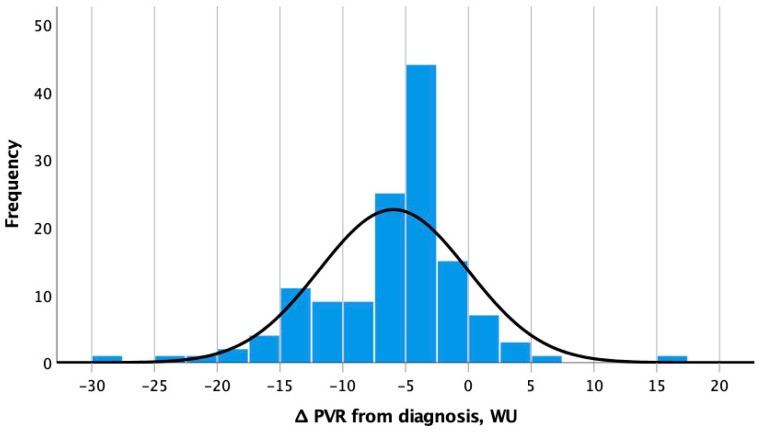
Distribution of decreases in pulmonary vascular resistance after 12 months of initial combination therapy. Abbreviation: PVR: pulmonary vascular resistance.

The median PVR reduction obtained in the overall population was −33% (IQR−60.7%; −22.0%) (−4.8 WU, IQR −9.0; −2.9 WU), resembling a normal distribution (Figure 1).

Patients with an upfront strategy including parenteral prostanoid plus one oral drug (Group-1) had −5.3 ± 6.2 WU (−50 ± 19%) reduction in PVR, patients with an upfront strategy including parenteral prostanoid plus double oral drug (Group-2) had −12.8 ± 5.9 WU (− 68 ± 17%) reduction in PVR, while patients with an add-on strategy including parenteral prostanoid after oral drugs (Group-3) had −3.9 ± 3.5 WU (−23 ± 19%) reduction in PVR. We decided not to further consider parenteral prostanoid monotherapy as this strategy was proposed at the very beginning (1998–2006) in very advanced patients in WHO class IV and instable hemodynamics, resulting in a heterogeneous treatment response.

A multivariate logistic analysis was used to identify baseline variables that were associated with a PVR reduction of more than −9.0 WU (i.e., the greater tertile of PVR changes). WHO functional class IV was independently associated with a reduced likelihood of achieving the greater reduction of PVR (OR 0.11, 95% C.I. 0.014–0.84, *p* < 0.03). Additionally, an upfront strategy including a parenteral prostanoid was independently associated with an increased likelihood of achieving the greater reduction of PVR compared with parenteral prostanoid as an add-on strategy to oral treatment (one oral drug plus parenteral prostanoid, OR 6.5, 95% C.I. 1.7–24, *p* < 0.006; two oral drugs plus parenteral prostanoid, OR 44, 95% C.I. 11–169, *p* = 0.0001).

**Figure 2:**
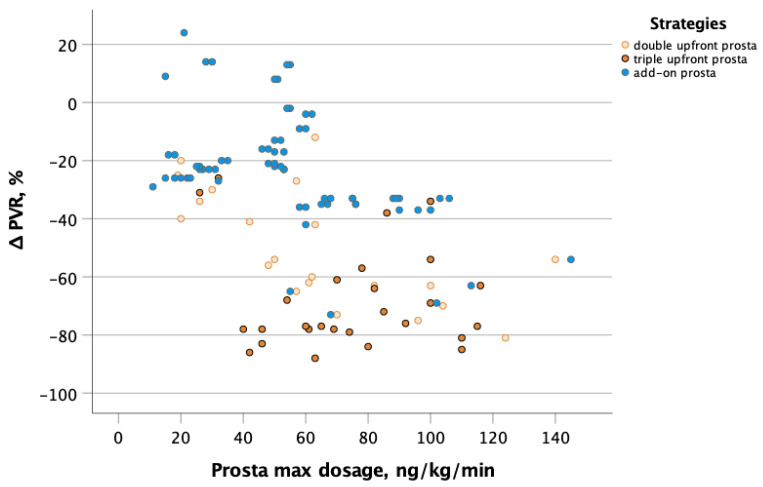
Correlation between changes in PVR and maximum dosage of parenteral prostanoids achieved at follow-up. Yellow circles indicate double upfront strategy including prostanoid plus one oral drug (Group 1); orange circles indicate triple upfront strategy including parenteral prostanoid plus double oral drug (Group 2); blue circles indicate add-on strategy including parenteral prostanoid after oral drugs (Group 3). Markedly, the upfront strategy including prostanoids is independently associated with reduction in PVR compared with an add-on strategy. The up-titration to higher dosage of prostanoid is more important for the add-on strategy and upfront with one oral drug strategy compared with the triple upfront. Abbreviation: PVR: pulmonary vascular resistance.

The maximum dosage of parenteral prostanoids achieved before the second evaluation was associated at univariate analysis with the increased odds of achieving the greater tertile of PVR changes, showing a 26% increase in the likelihood of PVR reduction greater than 9 WU for every additional increase of 10 ng/kg/min in dosage (OR 1.26, 95% C.I. 1.12–1.40, *p* < 0.001). However, in multivariate analysis, the treatment strategy emerged as more important (independently associated) than the maximum dosage. As shown in Figure 2, the up-titration to a higher dosage seems more important for the add-on strategy and upfront with one oral drug strategy compared with the triple upfront one.

**Figure 3:**
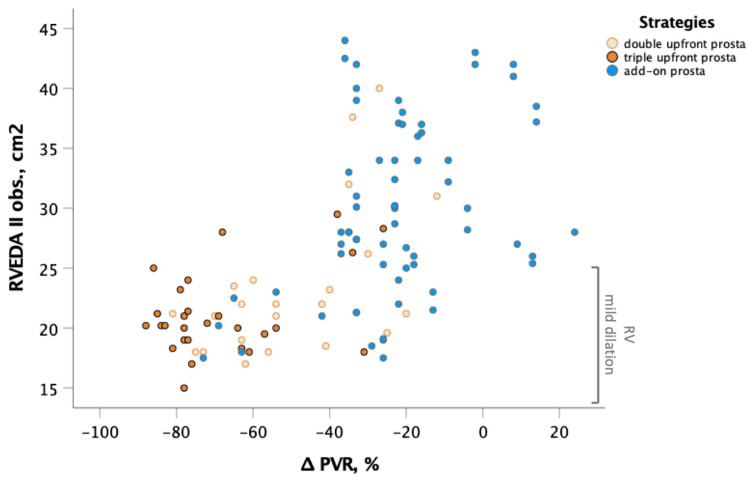
Correlation between changes in RVEDA and PVR at follow-up. Yellow circles indicate double upfront strategy including prostanoid plus one oral drug (Group 1); orange circles indicate triple upfront strategy including parenteral prostanoid plus double oral drug (Group 2); blue circles indicate add-on strategy including parenteral prostanoid after oral drugs (Group 3). Markedly more reverse remodeling in relation to more a important decrease in PVR was observed in patients treated with upfront combination therapy including prostanoid. Abbreviation: RVEDA: right ventricular end-diastolic area; PVR: pulmonary vascular resistance.

Figure 3 shows the tight relationship between PVR reduction and RVEDA at the second observation. The improvement to a mild RV dilation is clearly a function of the ability of the treatment strategy to achieve a greater reduction of PVR at last observation. The triple upfront combination with parenteral prostanoid emerged as the strategy with the higher likelihood of achieving a mild RV dilation compared with the add-on strategy to oral drugs (triple upfront: OR 13.6, 95% C.I. 4.2–43.9, *p* = 0.0001; one oral drug plus parenteral prostanoid: OR 5.1, 95% C.I. 1.9–13.5, *p* = 0.001).

**Figure 4:**
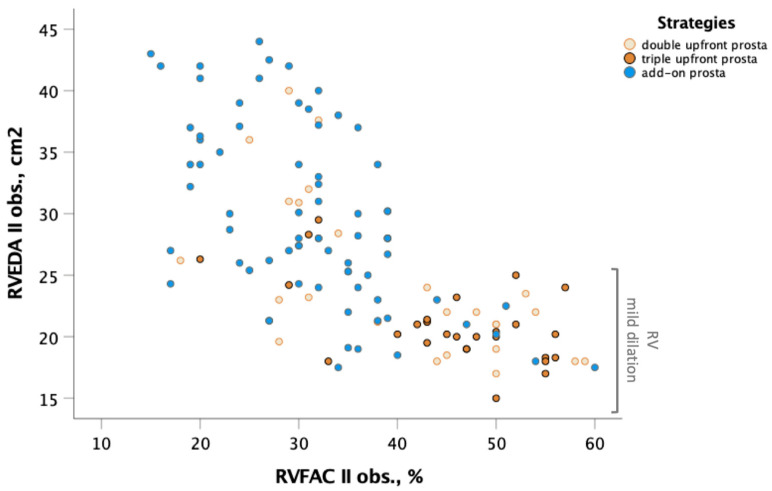
Correlation between RVEDA and RVFAC at follow-up. Yellow circles indicate double upfront strategy including prostanoid plus one oral drug (Group 1); orange circles indicate triple upfront strategy including parenteral prostanoid plus double oral drug (Group 2); blue circles indicate add-on strategy including parenteral prostanoid after oral drugs (Group 3). Markedly better recovery of RVFAC in relation to more important decrease in RVEDA was observed in patients treated with upfront combination therapy including prostanoid. Abbreviation: RVEDA: right ventricular end-diastolic area; RVFAC: right ventricular fractional area change.

Figure 4 illustrates an important concept. While different treatment strategies may lead to some degree of improvement in RV systolic function, it is unusual to have RVFAC within the normal range (RVFAC > 40%) unless a mild dilation of the RV has been achieved. This clinical condition is more common when an upfront triple combination with parenteral prostanoid has been started at the time of diagnosis, compared with other strategies involving parenteral prostanoids.

### 3.3. Changes in ESC/ERS Score and Determinants of Mantainence/Achievement of a Low-Risk Profile

At the second evaluation, a low-risk status was achieved in 75 patients (49.7%) according to the ERS/ESC score. Patients at low risk at the time of diagnosis were more likely to remain in a low-risk status, while a significant proportion of those at intermediate and high risk were unable to improve to a low-risk status.

At logistic analysis, PVR, RA area, and TAPSE at the time of diagnosis were independently associated with an ESC/ERS low-risk profile at the second evaluation (PVR: HR 0.91, 95% C.I. 0.85–0.97, *p* = 0.006; RA area: HR 0.89, 95% C.I. 0.84–0.95, *p* = 0.001; TAPSE: HR 1.14, 95% C.I. 1.06–1.35, *p* = 0.04). In particular, the likelihood of achieving an ESC/ERS low-risk status is increased by 14% for every unit increase in TAPSE at the time of diagnosis. On the other hand, each additional increase of one WU in PVR and one cm^2^ in RA area is associated with, respectively, an 8% and 11% decrease in the likelihood of achieving an ESC/ERS low-risk status. In other words, the greater the severity of PH at the time of diagnosis, in terms of PVR and RV remodeling, the higher the probability of treatment failure.

## 4. Discussion

In the present study, we showed that an upfront strategy including a parenteral prostanoid is associated with a greater reduction of PVR compared with parenteral prostanoid as an add-on to oral treatment. In addition, the triple upfront combination with parenteral prostanoid emerged as the treatment approach with the highest likelihood of achieving an ESC/ERS low-risk profile and greater RV reverse remodeling at follow-up.

Despite the growing number of targeted therapies available, PAH remains incurable [3,5,10,11,12,13,14] and clinical improvement and survival remain insufficient, especially when each of the approved PAH-specific therapies is used as monotherapy [15,16,17,18,19,20,21,22,23,24,25,26,27,28,29]. In the modern treatment era, the major advances in PAH treatment have mostly been related to the development of new strategies for combination therapy [16,30]. The combination of drugs targeting different pathways is not a new approach in PAH [1,29,30,31,32,33]. The previous therapeutic approach involved initiating monotherapy and adding other drugs in case of clinical deterioration or insufficient patient improvement (sequential combination therapy) [32,34,35,36,37,38,39,40]. Up-front combination therapy, as opposed to sequential therapy, is a more recent strategy that involves initiating combination therapy from the time of diagnosis.

The first trial to evaluate initial upfront combination therapy was the AMBITION trial, a multi-center international landmark study investigating the initial combination of the endothelin receptor antagonist (ambrisentan) and phosphodiesterase type 5 inhibitor (tadalafil) versus either drug alone, in 500 treatment-naïve PAH patients [1]. This was an event-driven morbidity and mortality study demonstrating a 50% reduction in the relative risk of clinical failure events with initial combination therapy compared with monotherapy. The results from the AMBITION trial led to an update of international guidelines [1]. Indeed, based on these results, experts agreed on double oral combination therapy in low and intermediate-risk patients and initial triple combination including parenteral prostacyclin in high-risk patients [16]. However, as previously mentioned, oral combination therapy achieves or maintains a low-risk status only in a minority of patients (35% according to the REVEAL 2.0 score or 43% according to the ERS/ESC score, with no high-risk patient achieving a low-risk status by either score and some patients continuing to experience disease progression [6,41].

Previous studies have shown that in patients with severe PAH, an even more aggressive treatment with the inclusion of a parenteral prostacyclin decreases PVR by an average of 65% and this favorable hemodynamic effect results in persistent clinical improvement, achievement of low-risk status and reverse remodeling of the right ventricle [6]. Sitbon et al. applied an initial triple combination of ERA, PDE5i, and intravenous epoprostenol in a small cohort of 19 patients with severe idiopathic PAH (WHO functional class III and IV), showing an unprecedented long-term improvement in WHO functional class, 6MWD, mPAP, and PVR after 4 months of therapy [42]. Similar results have been shown by D’Alto M. et al. in 21 patients with newly diagnosed high-risk idiopathic PAH, reporting sustained improvement in functional status and exercise capacity, and a fall in mPAP and PVR with a triple upfront combination of ERA, PDE5i, and subcutaneous treprostinil [9].

Even though these results have only been demonstrated in a small number of high-risk patient populations, these observations highlight the benefits of treating high-risk patients early with combination therapy including parenteral prostanoid and support the notion that this aggressive approach allows a greater decrease in PVR associated with improved risk status and right heart function [43].

However, despite international guidelines recommending the use of parenteral prostanoids for the treatment of PAH, the prognostic relevance of delayed treatment initiation is still debated, and the best timing remains a critical challenge.

A meta-analysis of six PAH RCTs [44] showed that a short-term delay of 12 to 16 weeks in newly diagnosed PAH patients has an adverse impact on functional capacity and outcome in the longer term. A previous study by our group has shown that late use of parenteral prostanoids is an important negative prognostic factor in PAH patients, emphasizing the need for early initiation in patients with severe PAH.

In keeping with previous data, our study showed that an early initiation of prostanoid therapy (upfront approach) was accompanied by a greater reduction in PVR and subsequent significant improvements in RV function and reverse remodeling with achievement of a low-risk status, compared to patients treated with parenteral prostanoid initiated as a second-line therapy (add-on to oral drug).

Interestingly, in our study, a high-risk profile at diagnosis is associated with a high likelihood of treatment failure. Indeed, WHO functional class IV was independently associated with a reduced likelihood of achieving a low-risk status. In other words, the greater the severity of PH at the time of diagnosis, the higher the probability of treatment failure.

These findings are particularly relevant given that, despite increasing awareness of the disease, there remains a significant delay from symptom onset to diagnosis. In fact, the majority of PAH patients have a very advanced disease at diagnosis, usually associated with NYHA FC III/IV and intermediate or high risk [10,44]. Survival rates for these patients, even in the modern treatment era, remain poor [9,43]. Consequently, the scientific community should focus on identifying the correct timing to initiate a more aggressive treatment in less advanced risk class, as the intermediate risk, where the therapeutic management remains challenging. Although larger multi-center studies are needed, our results provide strong preliminary evidence for the benefit of an initial combination regimen with parenteral prostanoid in incident patients with severe PAH compared with an add-on strategy.

## 5. Limitations

Our study is a retrospective observational single-center study with a relatively limited sample size. However, we believe that its results are convincing, as the study had original hemodynamic and echocardiography data with results analyzed with rigorous statistics.

The long enrollment time window over the past two decades may represent a second limitation, considering changes in treatment strategies provided by available guidelines. However, our center has over time emphasized the importance of a therapeutic strategy based on early aggressive treatment with parenteral prostanoids for patients with more advanced diseases. Therefore, our treatment approach remained basically unchanged over time for hemodynamically compromised patients. This may ensure a homogenous comparison of treatment groups.

Additional well-designed prospective studies with a larger sample size and including survival analysis are required to validate the present results in the future and to clarify the best timing for parenteral prostanoids in patients.

## 6. Conclusions

The present analysis supports the importance of significantly reducing PVR at short-term follow-up. The higher tertile of PVR reduction was shown to be tightly associated with the initial treatment strategy at the time of diagnosis, where the upfront combination of parenteral prostanoids resulted in being independently associated with an increased likelihood of important RV unloading and size reduction compared with the add-on strategy during follow-up.

As the likelihood of achieving an ESC/ERS low-risk status is reduced for patients with very high PVR at the time of diagnosis, especially when RV systolic function is reduced, the importance of choosing more aggressive treatment strategies since the beginning of the patient’s clinical history becomes mandatory to unload and allow a reverse remodeling of the RV.

This is clinically meaningful, since it provides more evidence on the importance of starting combination therapies including parenteral prostanoids since the time of diagnosis, especially for intermediate-risk patients who have a higher probability of huge PVR reduction compared with more advanced WHO class IV patients.

## Figures and Tables

**Table 1:** Baseline characteristics of the study population (prostanoid monotherapy included in the overall population).

		Total	Double Upfront (1)	Triple Upfront (2)	Add-On (3)	*p*
**Age**, years		55.6 ± 14.6	58 ± 11	59 ± 13	53 ± 15	ns (1–2)
						ns (1–3)
						ns (2–3)
**Gender**, F (%)		108 (71.5)	18 (69.2)	21 (75)	47 (65.3)	ns (1–2)
						ns (1–3)
						ns (2–3)
**Height**, cm		163 ± 9	166 ± 9.5	162 ± 9.5	164 ± 8	ns (1–2)
						ns (1–3)
						ns (2–3)
**Weight**, kg		68 ± 15	67 ± 15	70 ± 18	68 ± 14	ns (1–2)
						ns (1–3)
						ns (2–3)
**WHO class**		3 ± 0.4	3.0 ± 0.2	3.0 ± 0.2	3.2 ± 0.4	ns (1–2)
III		124 (82.1%)	25 (96.2%)	27 (96.4%)	55 (76.4%)	<0.01 (1–3)
IV		27 (17.9)	1 (3.8%)	1 (3.6%)	17 (23.6%)	<0.01 (2–3)
**6MWT**, m		308.4 ± 93	337.6 ± 70	330 ± 72.8	302 ± 106	ns (1–2)
						ns (1–3)
						ns (2–3)
**Hemodynamics**						
HR		82.4 ± 11	81.6 ± 15	78.5 ± 10	84 ± 10	ns (1–2)
						ns (1–3)
						ns (2–3)
PAPm, mmHg		57 ± 12	52.5 ± 10	61.4 ± 14.5	57.5 ± 11.6	0.02 (1–2)
						ns (1–3)
						ns (2–3)
PWP, mmHg		9.2 ± 3.5	9 ± 3.5	9.3 ± 4.5	9.3 ± 3.4	ns (1–2)
						ns (1–3)
						ns (2–3)
CI, L/min/m^2^		1.8 ± 0.4	2.0 ± 0.5	1.8 ± 0.4	1.8 ± 0.4	ns (1–2)
						ns (1–3)
						ns (2–3)
PVR, WU		16.4 ± 7	13.2 ± 5	18.3 ± 7	16.6 ± 7	0.02 (1–2)
						ns (1–3)
						ns (2–3)
RAP, mmHg		10 ± 5	10 ± 5.2	8.8 ± 5	10 ± 4	ns (1–2)
						ns (1–3)
						ns (2–3)
Compliance mL/mmHg		0.9 ± 0.4	1.2 ± 0.4	0.6 ± 0.2	0.9 ± 0.4	0.05 (1–2)
						ns (1–3)
						ns (2–3)
**Echocardiography**						
RVEDA, cm^2^		27.4 ± 6	26.5 ± 5	26.8 ± 4.7	28 ± 6.5	ns (1–2)
						ns (1–3)
						ns (2–3)
RVESA, cm^2^		18.3 ± 4.7	17.5 ± 4	17.8 ± 3.6	18.8 ± 5	ns (1–2)
						ns (1–3)
						ns (2–3)
RVFAC, %		33 ± 6.4	33.5 ± 7	33.4 ± 5.5	32.8 ± 6.6	ns (1–2)
						ns (1–3)
						ns (2–3)
TAPSE, mm		17 ± 3	17.5 ± 3.6	16.8 ± 2	17 ± 3	ns (1–2)
						ns (1–3)
						ns (2–3)
RA area, cm^2^		30.8 ± 8.7	29.5 ± 6.8	27.8 ± 6	32 ± 10	ns (1–2)
						ns (1–3)
						ns (2–3)
TR, grade						0.04 (1–2)
						ns (1–3)
						0.04 (2–3)
Mild		103 (68%)	18 (69.2%)	24 (85.7%)	47 (65.3%)	
Moderate		39 (26%)	6 (23.1%)	4 (14.3%)	20 (27.8%)	
Severe		9 (6%)	2 (7.7%)	0 (0%)	5 (6.9%)	
LV-Eid		1.7 ± 0.25	1.6 ± 0.2	1.6 ± 0.2	1.7 ± 0.3	ns (1–2)
						ns (1–3)
						ns (2–3)
LV-EIs		1.95 ± 0.5	1.8 ± 0.2	1.9 ± 0.3	2 ± 0.6	ns (1–2)
						ns (1–3)
						ns (2–3)
Pericardial Effusion		35 (23.1%)	6 (23.0%)	8 (28.5%)	15 (27.7%)	ns (1–2)
						ns (1–3)
						ns (2–3)
**ESC/ERS score**	interm	131 (86.8%)	25 (96.2%)	28 (100%)	59 (81.9%)	ns (1–2) 0.01 (1–3) 0.01 (2–3)
	high	20 (13.2%)	1 (3.8%)	0 (0%)	13 (18.1%)	
Months to prostanoid					7.8 ± 3	
Max dosage, ng/kg/min			59.6 ± 36	73.7 ± 31.4	52.8 ± 37	ns (1–2)
						ns (1–3)
						0.03 (2–3)

LEGEND: WHO: World Health Organization; 6MWT: non-encouraged 6-min walk test; HR: heart rate; PAPm: mean pulmonary arterial pressure; PWP: mean pulmonary wedge pressure; CI: cardiac index; PVR: pulmonary vascular resistance; RAP: right atrial pressure; RVEDA: right ventricle end-diastolic area; RVESA: right ventricle end-systolic area; RVFAC: right ventricle fractional area change; TAPSE: tricuspid annular plane systolic excursion; RA: right atrium area; TR: tricuspid regurgitation; LV-EId: left ventricular end-diastolic eccentricity index; LV-EIs: left ventricular end-systolic eccentricity index.

**Table 2:** Characteristics of the study population at the follow-up (prostanoid monotherapy included in the overall population).

		Total	Double Upfront (1)	Triple Upfront (2)	Add-On (3)	*p*
**WHO class**		2.5 ± 0.7	2.1 ± 0.4	2.1 ± 0.7	2.6 ± 0.7	ns (1–2)
						0.004 (1–3)
						0.002 (2–3)
I		4 (2.6%)	1 (3.8%)	3 (10.7%)	0 (0%)	
II		92 (60.9%)	21 (80.8%)	21 (75%)	38 (52.8%)	
III		36 (23.8)	4 (15.4%)	2 (7.1%)	23 (31.9%)	
IV		19 (12.6%)	0 (0%)	2 (7.1%)	11 (15.3%)	
**6MWT**, m		399.5 ± 110.8	440.5 ± 102	461.7 ± 78.5	375 ± 115	
						ns (1–2)
						0.02 (1–3)
						0.002 (2–3)
**Hemodynamics**						
HR		76 ± 11	79 ± 16.5	72 ± 6	78.6 ± 11	ns (1–2)
						ns (1–3)
						ns (2–3)
PAPm, mmHg		50.8 ± 21	40.6 ± 15.7	36.6 ± 21	58.3 ± 20	ns (1–2)
						0.001 (1–3)
						0.000 (2–3)
PWP, mmHg		9.7 ± 3.2	7.9 ± 3	9.5 ± 2.7	11.4 ± 3	ns (1–2)
						0.045 (1–3)
						ns (2–3)
CI, L/min/m^2^		2.5 ± 0.5	2.7 ± 0.4	2.9 ± 0.5	2.4 ± 0.5	ns (1–2)
						0.03 (1–3)
						0.000 (2–3)
PVR, WU		10 ± 6	7.5 ± 6	5.6 ± 4.8	12 ± 5	ns (1–2)
						0.001 (1–3)
						0.000 (2–3)
RAP, mmHg		6.7 ± 2.5	5.3 ± 1.5	5.4 ± 2	8 ± 2.8	ns (1–2)
						0.000 (1–3)
						0.000 (2–3)
Compliance mL/mmHg		1.7 ± 0.8	1.8 ± 1.3	1.9 ± 0.6	0.8 ± 0	0.05 (1–2)
						ns (1–3)
						ns (2–3)
**Echocardiography**						
RVEDA, cm^2^		27.3 ± 7	24 ± 6.5	21 ± 3.5	29 ± 7	ns (1–2)
						0.003 (1–3)
						0.000 (2–3)
RVESA, cm^2^		18 ± 7	14.8 ± 6	11.8 ± 3.7	20 ± 6.7	ns (1–2)
						0.001 (1–3)
						0.000 (2–3)
RVFAC, %		35 ± 11	40 ± 11	45 ± 9	31.5 ± 9	ns (1–2)
						0.000 (1–3)
						0.000 (2–3)
TAPSE, mm		18.6 ± 4	20.5 ± 4	22 ± 3	17.5 ± 3.5	ns (1–2)
						0.001 (1–3)
						0.000 (2–3)
RA Area, cm^2^		31.2 ± 9.6	27.8 ± 8.5	24 ± 6.7	33.7 ± 10	ns (1–2)
						0.02 (1–3)
						0.000 (2–3)
TR, grade						0.04 (1–2)
						ns (1–3)
						0.03 (2–3)
Mild		108 (71%)	21 (80.7%)	28 (100%)	52 (72.2%)	
Moderate		37 (25%)	5 (19.2%)	0 (0%)	17 (23.6%)	
Severe		6 (4%)	0 (0%)	0 (0%)	3 (4.1%)	
LV-EId		1.7 ± 0.4	1.5 ± 0.3	1.3 ± 0.25	1.8 ± 0.3	ns (1–2)
						0.000 (1–3)
						0.000 (2–3)
LV-EIs		1.9 ± 0.7	1.6 ± 0.5	1.45 ± 0.4	2.2 ± 0.7	ns (1–2)
						0.000 (1–3)
						0.000 (2–3)
Pericardial Effusion		21 (13.9%)	3 (11.5%)	2 (7.1%)	10 (13.9%)	ns (1–2)
						ns (1–3)
						ns (2–3)
**ESC/ERS score**	low interm	75 (49.7%)	19 (73.1%)	23 (82.1%)	26 (36.1%)	ns (1–2)
		58 (38.4%)	7 (26.9%)	3 (10.7%)	36 (50%)	0.000 (1–3)
	high	18 (11.9%)	0 (0%)	2 (7.1%)	10 (13.9%)	0.000 (2–3)

LEGEND: WHO: World Health Organization; 6MWT: non-encouraged 6-min walk test; HR: heart rate; PAPm: mean pulmonary arterial pressure; PWP: mean pulmonary wedge pressure; CI: cardiac index; PVR: pulmonary vascular resistance; RAP: right atrial pressure; RVEDA: right ventricle end-diastolic area; RVESA: right ventricle end-systolic area; RVFAC: right ventricle fractional area change; TAPSE: tricuspid annular plane systolic excursion; RA: right atrium area; TR: tricuspid regurgitation; LV-EId: left ventricular end-diastolic eccentricity index; LV-EIs: left ventricular end-systolic eccentricity index.

## Data Availability

Due to privacy and ethical concerns, the data cannot be made available. However, a specific request might be considered by the authors.

## Abbreviations

6MWT	6-min walk test
CO	cardiac output
CI	cardiac index
ERA	endothelin receptor antagonists
IPAH	idiopathic pulmonary arterial hypertension
LV	left ventricular
LV-EId	left ventricular diastolic eccentricity index
LV-EIs	left ventricular systolic eccentricity index
mPAP	mean pulmonary artery pressure
PAH	pulmonary arterial hypertension
PDE5i	phosphodiesterase-5 inhibitors
PVR	pulmonary vascular resistance
PWP	pulmonary wedge pressure
RA	right atrium
RAP	right atrial pressure
RHC	right heart catheterization
RV	right ventricular
WHO	World Health Organization
